# Serum amylase elevation is associated with adverse clinical outcomes in patients with coronavirus disease 2019

**DOI:** 10.18632/aging.203653

**Published:** 2021-10-29

**Authors:** Ganxun Li, Tongtong Liu, Guannan Jin, Tianhong Li, Junnan Liang, Qian Chen, Lin Chen, Wei Wang, Yuwei Wang, Jia Song, Huifang Liang, Chuanhan Zhang, Peng Zhu, Wanguang Zhang, Zeyang Ding, Xiaoping Chen, Bixiang Zhang, Tongji Multidisciplinary Team for Treating COVID-19 (TTTC)

**Affiliations:** 1 Hepatic Surgery Center, and Hubei Key Laboratory of Hepato-Pancreatic-Biliary Diseases, National Medical Center for Major Public Events, Tongji Hospital, Tongji Medical College, Huazhong University of Science and Technology, Wuhan 430030, Hubei, China; 2 Department of Anesthesiology, National Medical Center for Major Public Events, Tongji Hospital, Tongji Medical College, Huazhong University of Science and Technology, Wuhan 430030, Hubei, China; 3 Department of Nephrology, Union Hospital, Tongji Medical College, Huazhong University of Science and Technology, Wuhan 430030, Hubei, China; 4 Nursing Department, Wuhan Children’s Hospital, Tongji Medical College, Huazhong University of Science and Technology, Wuhan 430030, Hubei, China; 5 Department of Gastroenterology, National Medical Center for Major Public Events, Tongji Hospital, Tongji Medical College, Huazhong University of Science and Technology, Wuhan 430030, Hubei, China

**Keywords:** hyperamylasemia, COVID-19, pancreatic injury, multiorgan dysfunction, mortality

## Abstract

Objective: Hyperamylasemia was found in a group of patients with COVID-19 during hospitalization. However, the evolution and the clinical significance of hyperamylasemia in COVID-19, is not well characterized.

Design: In this retrospective cohort study, the epidemiological, demographic, laboratory, treatment and outcome information of 1,515 COVID-19 patients with available longitudinal amylase records collected from electronic medical system were analyzed to assess the prevalence and clinical significance of hyperamylasemia in this infection. Associated variables with hyperamylasemia in COVID-19 were also analyzed.

Results: Of 1,515 patients, 196 (12.9%) developed hyperamylasemia, among whom 19 (1.3%) greater than 3 times upper limit of normal (ULN) and no clinical acute pancreatitis was seen. Multivariable ordered logistic regression implied older age, male, chronic kidney disease, several medications (immunoglobin, systemic corticosteroids, and antifungals), increased creatinine might be associated with hyperamylasemia during hospitalization. Restricted cubic spline analysis indicated hyperamylasemia had a J-shaped association with all-cause mortality and the estimated hazard ratio per standard deviation was 2.85 (2.03-4.00) above ULN. Based on the multivariable mixed-effect cox or logistic regression model taking hospital sites as random effects, elevated serum amylase during hospitalization was identified as an independent risk factor associated with in-hospital death and intensive complications, including sepsis, cardiac injury, acute respiratory distress syndrome, and acute kidney injury.

Conclusions: Elevated serum amylase was independently associated with adverse clinical outcomes in COVID-19 patients. Since early intervention might change the outcome, serum amylase should be monitored dynamically during hospitalization.

## INTRODUCTION

Owing to the emergence of Severe Acute Respiratory Syndrome Coronavirus 2 (SARS-CoV-2), an outbreak of coronavirus disease 2019 (COVID-19) has violently spread almost all over the world. Carrying significant morbidity and mortality worldwide and posing an enormous threat to human beings, COVID-19 has developed into a global pandemic [1].

The COVID-19 pneumonia was primarily featured by fever, cough, fatigue, the cause of critical and even lethal lower respiratory tract infection, as well as extrapulmonary manifestations [2, 3]. An endeavor has been made by researchers to reveal the epidemiological, virological, and clinical characteristics of this pandemic [4–6]. However, most of the studies lay stress on illustrating respiratory symptoms, common complications, and significant risk factors of severe or deceased cases [7–9], while some non-classical but not insignificant morbidities or acute organ injury have been overlooked. For instance, our previous study indicated that a mild elevation of liver chemistries is most commonly found in patients with COVID-19 [10]. In addition, a portion of COVID-19 patients with serum amylase level elevation were observed in our clinical practice.

Previous studies attributed amylase abnormality to potential pancreatic damage caused by COVID-19 infection [11], which overemphasize the pancreatic source of amylase and overlook other possibility leading to hyperamylasemia. Studies of decades discovered that serum amylase levels depend on a balance between secretion and clearance [12]. Although recent research had identified its novel roles acting as promising diagnostic, therapeutic and prognostic biomarker applied to infection, cancer, and wound healing [13–15], none of previous studies with sufficient patients had evaluated the robust role of serum amylase in the COVID-19 progression. In addition, attribution elevated amylase in patients with COVID-19 to pancreatic injury is still a highly controversial issue [16, 17]. To address this concern, we designed and conducted this retrospective study to reveal the temporal and distributional patterns of serum hyperamylasemia in COVID-19 patients with a focus on its clinical significance and determinants.

## MATERIALS AND METHODS

### Study design and ethics

We conducted this retrospective study to investigate the clinical characteristics and outcomes of inpatients with COVID-19 who admitted to Tongji Hospital, a tertiary hospital designed by Chinese government for hospitalization of COVID-19 patients. All consecutive patients with a diagnosis of COVID-19 hospitalized in 3 different sites (Main District, Sino-French New City Branch, and Optical Valley Branch) of Tongji Hospital were included in this study. The study protocol was reviewed and approved by the Institutional Review Board of Tongji Hospital of Tongji Medical College, Huazhong University of Science and Technology (Grant No. TJ-IRB-20200207).

### Patient selection

**Figure 1:**
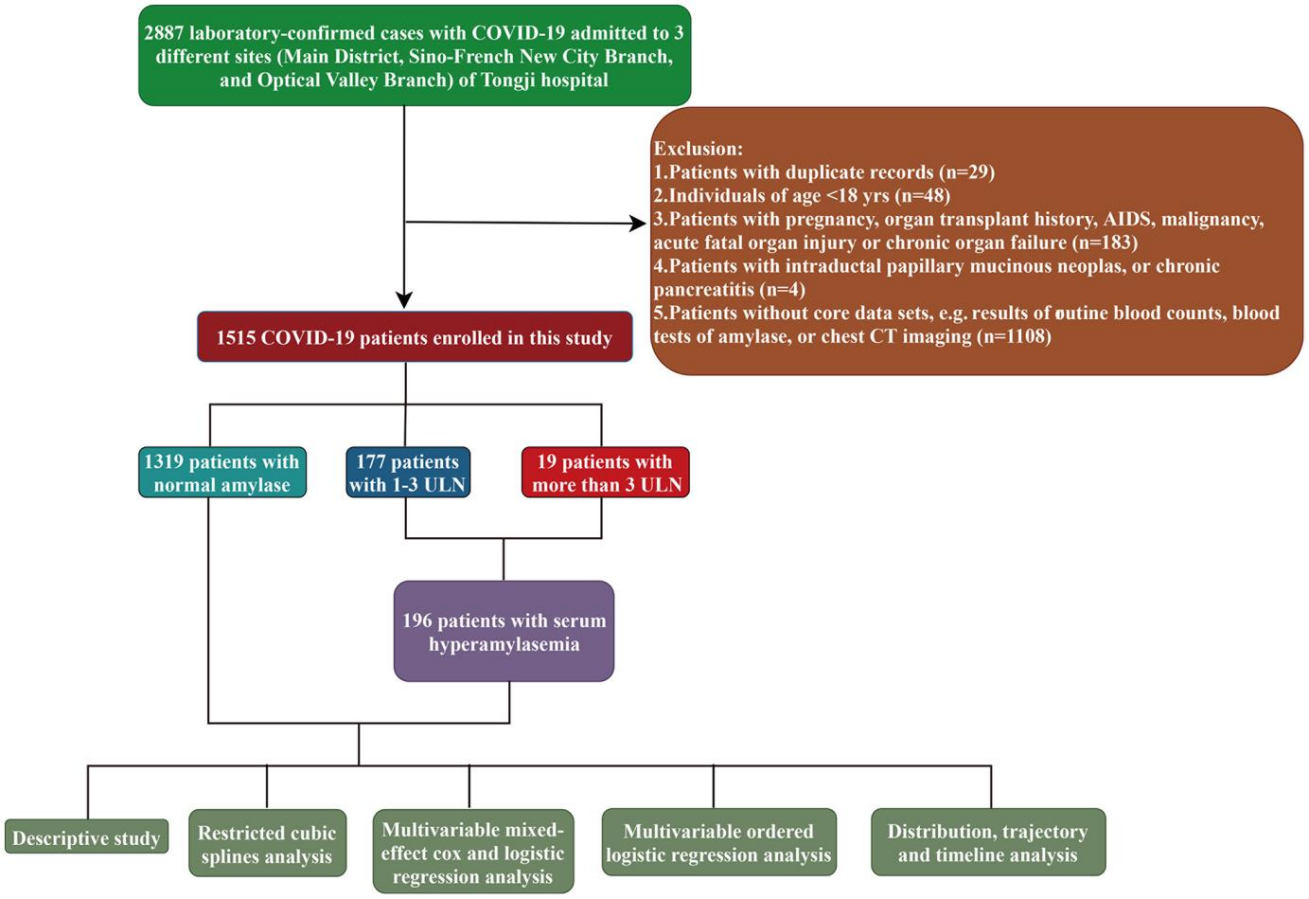
The flowchart showing enrollment of participants in this study.

COVID-19 was diagnosed according to ‘Clinical management of severe acute respiratory infection when novel coronavirus (nCoV) infection is suspected: interim guidance’ published by World Health Organization. Virological diagnosis was established by a positive result of transcription-polymerase chain reaction (RT-PCR) assay for SARS-CoV-2 nucleic acid from the nasal and pharyngeal swab specimens. We used the following inclusion and exclusion criteria to select patients. All consecutive virological-diagnosed COVID-19 patients who were subject to serious illness sufficient to admission in any branch of Tongji hospital between 18 January 2020 and 18 March 2020 were included in this study. Patients with any positive of the exclusion criteria as following were excluded: under 18 yr-old; absent of or with a duplicate medical record; a lack of core data (results of routine blood counts, blood tests of amylase, or chest CT imaging); with pregnancy, organ transplant history, AIDS, malignancy, acute fatal organ injury (e.g., acute myocardial infarction, acute coronary syndrome, acute pulmonary embolism, or acute stroke) or chronic organ failure (e.g., decompensated cirrhosis, decompensated chronic renal insufficiency, or severe congestive heart failure); with intraductal papillary mucinous neoplasms, or chronic pancreatitis. The detailed inclusion and exclusion criteria were summarized in Figure 1.

### Data collection and patient follow-up

All data were extracted from the Tongji Cloud Hospital Information System (an electronic database of medical records). Three authors independently collected and double-checked the clinical demographic information, pre-existing morbidities, symptoms and vital signs, laboratory examinations, radiological findings at admission, treatment and clinical outcomes during hospitalization of the patients with standardized forms. Before data extraction, personal identification information (e.g., name, medical ID) were removed and anonymized with an electronic code out of patient privacy protection. The pre-existing comorbidities were recorded by the physicians as part of routine clinical care based on patient self-report and medical history, and categorized according to ICD-10 coding.

All patients were followed up from the day at admission until they reached definite primary endpoint (discharge from or death in the hospital) and no patient was lost to follow up. The median follow-up time was 21 [IQR, 12-33] days. On 23 April 2020, the final date of follow up, we finished data extraction and started analysis and all patients reach primary endpoint.

### Outcomes and definitions

The observation period of the time-vary variables was the duration between hospital admission and composite endpoint. The patients who presented with a peak value of serum amylases above ULN during illness were categorized into ESA (elevated serum amylase) group, while their counterparts with normal peak values into non-ESA group. The primary outcome was in-hospital death and secondary outcomes were the incidences of common critical complications during hospitalization, such as SARS-CoV-2 related acute respiratory distress syndrome (ARDS), acute kidney injury (AKI), sepsis, acute cardiac injury, and disseminated intravascular coagulation (DIC).

The diagnosis of hyperamylasemia or elevated serum amylase is established with an elevated serum amylase beyond ULN. The ULN of serum amylase (115 U/L) was determined by determined at the clinical laboratory of Tongji Hospital. Diagnosis of acute pancreatitis was based on Chinese guidelines for the management of acute pancreatitis (Shenyang, 2019) [18]. In detail, the diagnosis of acute pancreatitis requires at least two of the following three features: (1) abdominal pain consistent with acute pancreatitis (acute onset of a persistent, severe, epigastric pain often radiating to the back); (2) serum lipase activity (or amylase activity) at least three times greater than the upper limit of normal; and (3) characteristic findings of acute pancreatitis on contrast-enhanced computed tomography (CECT) and less commonly magnetic resonance imaging (MRI) or abdominal ultrasonography.

The definition of ARDS and sepsis was according to the interim guidance of WHO. We defined the acute kidney injury according to an elevation in serum creatinine (SCr) by ≥0.3 mg/dl (≥26.5 μmol/l) within 48 hours or SCr to ≥1.5 times baseline within the prior 7 days.

Cardiac injury was defined by concentrations of any cardiac biomarkers (e.g., cardiac troponin I (cTNI), cardiac troponin T (cTNT), or high sensitivity cardiac troponin I (hs-cTNI)) higher than the upper limit of the normal range [9]. The diagnosis of DIC was established on the basis of the criteria illustrated in the International Society on Thrombosis and Hemostasis [19]. Definition of hyperlipidemia was according to 2019 ESC/EAS Guidelines for the management of dyslipidaemias [20].

### Public RNA-Seq data

RNA-Seq dataset originated from the Genotype Tissue Expression Project (GTEx), corresponding to 8,555 samples from 31 normal human tissues, was downloaded from UCSC Xena [21]. Using Toil, UCSC’s pipeline architecture, the expression of total genes was recomputed to create a consistent meta-analysis of the dataset free of computational batch effects [22]. Transcripts Per Kilobase of exon model per Million mapped reads (TPM) values of *ACE2* and *TMPRSS2* was extracted, and log2(TPM+0.001) transformed.

### Statistical analysis

Continuous variables were provided as median (interquartile range [IQR]) and compared with independent group t tests or Mann-Whitney test depending on whether the data were normal distribution. Categorical data were presented as absolute count (percentage) and were compared with the Chi-square test or Fisher exact test. Distribution of peak amylase by the mortality of COVID-19 was evaluated by kernel density estimation and the dynamic changes of peak amylase by the mortality of COVID-19 were predicting using Generalized Additive Models (GAM) [23]. Adjusted hazard ratios (HR) or odds ratios(OR) and 95% confidence interval (95% CI) were calculated based on multivariable mixed-effect Cox proportional hazard [24] or mixed-effect logistic regression model [25] taking sites (hospital branches) as random effects by adjusting for age > 65-yr-old, sex, body mass index, gastrointestinal symptoms (including anorexia, nausea or vomiting, diarrhea, abdominal pain), comorbidities (hypertension, diabetes, coronary artery disease, cerebrovascular disease, chronic kidney disease, chronic liver disease, and chronic pulmonary disease), and severity of COVID-19 in the crude cohorts. Kaplan-Meier survival analysis was used to illustrate the cumulative rate of in-hospital mortality [26]. Ordered logistic regression analysis with the negative log-log link acting as the increasing function was also conducted to reveal the correlation of baseline clinical characteristics and medications happened before peaking of amylase in the longitudinal cohort, where serum amylase was trichotomized. Restricted cubic spline analysis with three knots at the 5th, 50th, and 95th centiles, which could make model flexible, was used to evaluate whether the correlation between serum amylase and COVID-19 mortality was linear with the reference value (OR=1) at 115 U/L for serum amylase concentration [27]. Interaction contrast ratios (ICR) were calculated to assess the additive interaction between serum amylase and common clinical characteristics in the cox regression [28]. No variables with missing data were used for aforementioned regression analysis so there is no need to made imputation for the missing data. All statistical tests were two-sided and statistical significance was taken as *p*<0.05. All statistical analyses were conducted using R version 3.5.1 (R Foundation for Statistical Computing, Vienna, Austria).

### Ethics approval

The study protocol was reviewed and approved by the Institutional Review Board of Tongji Hospital of Tongji Medical College, Huazhong University of Science and Technology (Grant No. TJ-IRB-20200207).

### Data availability statement

Data are available on reasonable request.

## RESULTS

### Descriptions of cohort

Altogether 1515 laboratory-confirmed COVID-19 patients were analyzed in this study, among whom 196 presented with serum amylase level elevation, 19 (19/196, 9.7%) greater than 3-fold of ULN (>3ULN; >345 U/L) during hospitalization. None of patients with serum amylase higher than 3-fold of ULN developed abdominal pain during hospitalization. According to Revised Atlanta Classification [16, 29], the clinical manifestations and limited available abdominal imaging examinations in our study indicated that no acute pancreatitis diagnosis was established. The clinical characteristics and outcomes of those with serum amylase >345U/L were presented in Supplementary Table 1.

Compared with individuals in non-ESA group (Table 1), those with elevated serum amylases levels were older (median [IQR], 66 [56, 73] vs 60 [49, 68] years) and with a larger proportion of males (66.8% vs 45.9%). Cough, fatigue and dyspnea were significantly more prevalent in ESA group than and in non-ESA group, while no significant difference was noted between the two groups for gastrointestinal symptoms (any symptoms of anorexia, nausea or vomiting, diarrhea, and abdominal pain) (41.3% vs 41.8%). Their baseline characteristics, pre-existing morbidities, clinical symptoms and vital signs at admission were provided in Table 1.

**Table 1:** Basic characteristics of COVID-19 patients with or without serum amylase abnormality.

	**Characteristic**		**Serum amylase level**		
		**Overall**	**Elevated**	**Normal**	***P* value**
		**N=1515**	**N=196**	**N=1319**	
	Age- yr	61 [49, 69]	66 [56, 73]	60 [49, 68]	<0.001
	Age≥ 65	602 (39.7)	114 (58.2)	488 (37.0)	<0.001
	Male	737 (48.6)	131 (66.8)	606 (45.9)	<0.001
	BMI	23.9 [22.1, 25.7]	23.7[21.8, 25.5]	23.9 [22.2, 25.7]	0.12
	Time from illness onset to hospital admission, days	14 [9, 24]	11.50 [7, 18]	15 [9, 25.6]	<0.001
	Severe pneumonia (NHC)^*^	652 (43.0)	112 (57.1)	540 (40.9)	<0.001
Signs and symptoms					
	Fever	1132 (74.7)	155 (79.1)	977 (74.1)	0.16
	Cough	1086 (71.7)	157 (80.1)	929 (70.4)	0.01
	Fatigue	449 (29.6)	72 (36.7)	377 (28.6)	0.03
	Chest pain	103 (6.8)	10 (5.1)	93 (7.1)	0.39
	Gastrointestinal symptoms^**^	633 (41.8)	81 (41.3)	552 (41.8)	0.95
	Dyspnea	524 (34.6)	84 (42.9)	440 (33.4)	0.01
	Myalgia	247 (16.3)	33 (16.8)	214 (16.2)	0.91
	Ascites	3 (0.2)	1 (0.5)	2 (0.2)	0.847
Vital signs					
	Respiratory rate, breaths per minute	20 [20, 22]	20 [20, 24]	20 [20, 22]	0.01
	Pulse, beat per minute	85 [78, 97]	86 [78, 99]	85 [78, 97]	0.17
	Mean arterial pressure, mmHg	97 [89, 105]	97 [90, 104]	97 [89, 105]	0.47
	Percutaneous oxygen saturation	97 [95, 98]	96 [92, 98]	97 [95, 98]	<0.001
Pre-existing comorbidity					
	Chronic liver disease	121 (8.0)	21 (10.7)	100 (7.6)	0.17
	Cardio-cerebrovascular metabolic diseases^$^	542 (35.8)	93 (47.4)	449 (34.0)	<0.001
	Chronic pulmonary disease^#^	91 (6.0)	15 (7.7)	76 (5.8)	0.38
	Chronic kidney disease	57 (3.8)	18 (9.2)	39 (3.0)	<0.001

1. Data were provided as number (percentage), median (interquartile range).

2. Serum Amylase Level, in a healthy individual, a normal blood amylase level ranges from 0-115 units per liter (U/L) in our hospital. The normal group included patients with normal serum amylase level, while the elevated group with serum amylase level >115U/L.

3. Severe pneumonia (NHC)^*^, the illness severity was classified according to Guidance for Corona Virus Disease 2019 (6/7th edition) released by the National Health Commission of China; Gastrointestinal symptoms^**^, including anorexia, nausea or vomiting, diarrhea, abdominal pain; Chronic pulmonary diseases^#^ contain chronic obstructive pulmonary disease, asthma, bronchiectasis and pulmonary fibrosis; Cardio-cerebrovascular metabolic diseases^$^ includes hypertension, cardiovascular disease, cerebrovascular disease, diabetes.

4. Abbreviations: COVID-19, coronavirus disease 2019; SMD, standard mean difference; BMI, body mass index, which was calculated as weight in kilograms divided by height in meters squared.

5. Comorbidities diagnoses are established by medical history and classified according to ICD-10 coding. These include, but are not limited to, those shown in the table.

Patients in ESA group presented with significantly more frequent and prominent abnormalities in laboratory findings than in non-ESA group (Table 2), including complete blood count, coagulation function, liver and kidney function, myocardial injury marker, and inflammatory parameters. Moreover, patients with hyperamylasemia were more prone to presented with bilateral involvement in radiological imaging than those with normal serum amylases (93.9% vs 83.6%). In addition, four of 19 patients with serum amylase greater than 3 times of ULN underwent abdominal CT imaging and no evident abnormality were found by experienced radiologists.

**Table 2:** Laboratory findings and radiological features of COVID-19 patients with or without serum amylase abnormality.

**Laboratory findings**		**Normal range**		**Serum amylase level**		***P***
			**Overall**	**Elevated**	**Normal**	
			**N=1515**	**N=196**	**N=1319**	
Blood routine						
	Leukocyte count, ×10^9^ per L	3·5-9·5	5.86 [4.52, 7.70]	6.50 [4.74, 8.87]	5.77 [4.50, 7.47]	<0.001
	>9·5		188/1515 (12.4)	45/196 (23.0)	143/1319 (10.8)	<0.001
	Neutrophil count, ×10^9^ per L	1·8-6·3	3.79 [2.66, 5.46]	4.93 [3.16, 7.35]	3.64 [2.61, 5.28]	<0.001
	>6·3		274/1515 (18.1)	66/196 (33.7)	208/1319 (15.8)	<0.001
	Lymphocyte count, ×10^9^ per L	1·1-3·2	127.00 [115.00, 138.00]	129.00 [116.00, 139.25]	127.00 [115.00, 138.00]	0.61
	<1·1		340/1515 (22.4)	46/196 (23.5)	294/1319 (22.3)	0.78
	Platelet count, ×10^9^ per L	125-350	1.19 [0.78, 1.64]	0.84 [0.60, 1.23]	1.24 [0.85, 1.68]	<0.001
	<125		689/1515 (45.5)	132/196 (67.3)	557/1319 (42.2)	<0.001
Coagulation function						
	Prothrombin time, s	11·5-14·5	13.80 [13.20, 14.40]	14.00 [13.40, 15.00]	13.70 [13.12, 14.30]	<0.001
	>14·5		321/1505 (21.3)	72/195 (36.9)	249/1310 (19.0)	<0.001
	D-dimer, ug/ml FEU	<0·5	0.65 [0.29, 1.63]	1.38 [0.58, 3.36]	0.58 [0.26, 1.43]	<0.001
	>0·5		851/1494 (57.0)	152/193 (78.8)	699/1301 (53.7)	<0.001
Liver function						
	Aspartate aminotransferase, U/L	≤40	25.00 [18.00, 37.00]	32.00 [22.00, 47.25]	24.00 [18.00, 35.00]	<0.001
	>40		317/1515 (20.9)	70/196 (35.7)	247/1319 (18.7)	<0.001
	Alanine aminotransferase, U/L	≤41	22.00 [14.00, 38.00]	25.00 [16.75, 38.00]	22.00 [14.00, 38.00]	0.02
	>41		342/1515 (22.6)	45/196 (23.0)	297/1319 (22.5)	0.96
	Total bilirubin, μmol/L	≤21	8.70 [6.40, 12.10]	9.30 [7.00, 13.90]	8.60 [6.40, 11.95]	0.01
	>21		73/1515 (4.8)	22/196 (11.2)	51/1319 (3.9)	<0.001
	Direct bilirubin, μmol/L	≤8·0	3.70 [2.70, 5.20]	4.40 [3.10, 6.40]	3.60 [2.70, 5.05]	<0.001
	>8·0		128/1515 (8.4)	34/196 (17.3)	94/1319 (7.1)	<0.001
Hyperlipidemia*			100 (6.6)	10 (5.1)	90 (6.8)	0.452
Renal function						
	Creatinine,μmol/L	45-84	69.00 [57.00, 83.00]	80.00 [64.75, 100.25]	67.00 [56.00, 81.00]	<0.001
	>84		354/1515 (23.4)	83/196 (42.3)	271/1319 (20.5)	<0.001
	Blood urea nitrogen, mmol/L	1·7-8·3	4.50 [3.50, 5.85]	5.50 [4.00, 7.95]	4.40 [3.50, 5.60]	<0.001
	>8·3		140/1515 (9.2)	46/196 (23.5)	94/1319 (7.1)	<0.001
	eGFR, ml/min/1·73m2	>90	92.30 [73.55, 103.10]	79.00 [46.40, 101.10]	92.90 [75.57, 103.40]	<0.001
	<90		691/1507 (45.9)	115/195 (59.0)	576/1312 (43.9)	<0.001
Cardiac markers						
	Creatinine kinase, U/L	≤190	61.00 [41.00, 99.00]	74.00 [45.00, 146.00]	60.00 [40.00, 96.00]	<0.001
	>190		119/1230 (9.7)	36/169 (21.3)	83/1061 (7.8)	<0.001
	High-sensitivity cardiac troponin I	≤15·6	3.80 [1.00, 9.60]	7.60 [3.05, 25.80]	3.30 [1.00, 8.07]	<0.001
	>15·6		211/1249 (16.9)	57/171 (33.3)	154/1078 (14.3)	<0.001
	N-terminal pro-brain natriuretic peptide	<486	106.00 [35.00, 325.25]	259.00 [76.00, 1209.00]	91.00 [31.00, 282.00]	<0.001
	>486		233/1252 (18.6)	63/171 (36.8)	170/1081 (15.7)	<0.001
Inflammation makers						
	High sensitivity C-reactive protein, mg/L	<10	12.30 [2.00, 57.80]	48.50 [12.45, 105.85]	10.10 [1.70, 47.95]	<0.001
	≥10		808/1511 (53.5)	149/196 (76.0)	659/1315 (50.1)	<0.001
	Interleukin-6, pg/ml	<7.0	5.31 [2.08, 21.18]	15.02 [4.99, 55.25]	4.67 [1.91, 18.50]	<0.001
	≥7.0		633/1402 (45.1)	126/182 (69.2)	507/1220 (41.6)	<0.001
Chest CT on admission						
	Bilateral lesion		1287 (85.0)	184 (93.9)	1103 (83.6)	<0.001

1. Data were provided as number (percentage), median (Interquartile range).

2. The normal range of laboratory parameters were based on Tongji hospital and might vary in different hospitals.

Individuals with serum amylase level elevation during hospitalization require more intensive integrated in-hospital treatment to manage COVID-19 (Table 3). Overall, individuals in EAS group censored a more frequent requirement for antibiotics (90.3% vs 74.1%), antifungal drugs (11.7% vs 2.7%), systemic corticosteroids (75.0% vs 43.7%), renal replacement therapy (15.8% vs 1.7%), as well as administration of mechanical ventilation (40.3% vs 13.6%), either non-invasively or invasively.

**Table 3:** Treatments in hospital of COVID-19 patients with or without serum amylase abnormality.

			**Serum amylase level**		***P* value**
		**Overall**	**Elevated**	**Normal**	
		**N=1515**	**N=196**	**N=1319**	
Treatments in hospital					
	Antiviral therapy	1429 (94.3)	189 (96.4)	1240 (94.0)	0.23
	Antibiotics	1154 (76.2)	177 (90.3)	977 (74.1)	<0.001
	Antifungal drugs	58 (3.8)	23 (11.7)	35 (2.7)	<0.001
	NSAIDs	203 (13.4)	46 (23.5)	157 (11.9)	<0.001
	Systemic corticosteroids	723 (47.7)	147 (75.0)	576 (43.7)	<0.001
	Intravenous immunoglobin	583 (38.5)	120 (61.2)	463 (35.1)	<0.001
	Traditional Chinese Medicine treatment	1299 (85.7)	167 (85.2)	1132 (85.8)	0.90
	Proton pump inhibitor	694 (45.8)	132 (67.3)	562 (42.6)	<0.001
	somatostatin analog	3 (0.2)	1 (0.5)	2 (0.2)	0.847
	Fasting or parenteral nutrition	251 (16.6)	62 (31.6)	189 (14.3)	<0.001
Administration of mechanical ventilation		258 (17.0)	79 (40.3)	179 (13.6)	<0.001
	Non-invasive	127 (8.4)	27 (13.8)	100 (7.6)	0.01
	Invasive	131 (8.6)	52 (26.5)	79 (6.0)	<0.001
Admission to intensive care unit		156 (10.3)	63 (32.1)	93 (7.1)	<0.001
Renal replacement therapy		54 (3.6)	31 (15.8)	23 (1.7)	<0.001
Extracorporeal membrane oxygenation		18 (1.2)	12 (6.1)	6 (0.5)	<0.001

Data were provided as number (percentage), median (interquartile range).

### Complications and in-hospital mortality in COVID-19 patients

As presented in Table 4, COVID-19 patients with elevated serum amylases usually developed complications of acute kidney injury, ARDS, acute heart failure, and cardiac injury, the incidence was significantly higher in those with normal serum amylases (34.2% vs 5.1%, 45.9% vs 16.7%, 43.9% vs 13.1%, and 56.1% vs 21.7%), respectively.

**Table 4:** Clinical outcomes of COVID-19 patients with or without serum amylase abnormality.

			**Serum amylase level**		***P* value**
		**Overall**	**Elevated**	**Normal**	
		**N=1515**	**N=196**	**N=1319**	
Clinical outcomes					
In-hospital death		118 (7.8)	46 (23.5)	72 (5.5)	<0.001
	Duration from illness onset to death, days	25 [19, 36]	29 [21, 41]	25 [19, 34]	0.11
Hospital discharge		1397 (92.2)	150 (76.5)	1247 (94.5)	<0.001
	Duration from illness onset to discharge, days	42 [32, 54]	45 [34, 56]	42 [32, 54]	0.06
Complications					
Acute kidney injury		134 (8.8)	67 (34.2)	67 (5.1)	<0.001
Acute respiratory distress syndrome		310 (20.5)	90 (45.9)	220 (16.7)	<0.001
Acute heart failure		217 (17.3)	75 (43.9)	142 (13.1)	<0.001
Cardiac injury		330 (26.4)	96 (56.1)	234 (21.7)	<0.001
Sepsis		174 (11.5)	62 (31.6)	112 (8.5)	<0.001
Disseminated intravascular coagulation		77 (5.2)	41 (21.2)	36 (2.8)	<0.001

Data were provided as number (percentage), median (interquartile range).

Overall 118 in-hospital death occurred in our follow-up period, among whom 46 (23.5%) presented with abnormalities in serum amylase versus 72 (5.5%) with normal range. Unfortunately, 16 (84.2) patients with serum amylase higher than 3-fold of ULN were admitted in intensive care unit and 78.9% (15/19) died of COVID-19 in hospital.

### Associations between elevated serum amylase and mortality, as well as secondary outcomes

**Figure 2:**
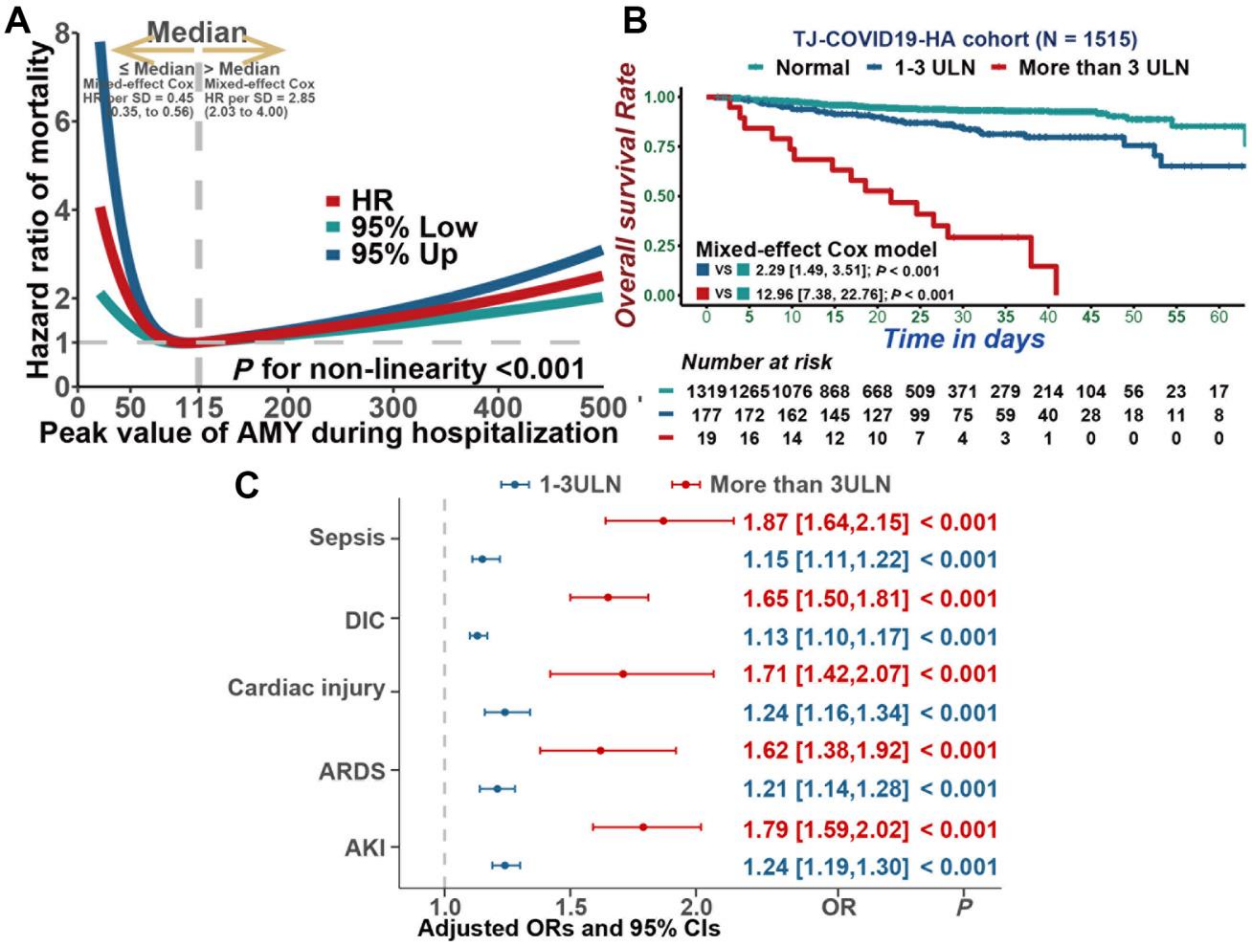
**Associations between serum amylase and mortality, as well as secondary outcomes.** (**A**) Restricted cubic splines analysis illustrated the association between serum amylase and all cause mortality. (**B**) Kaplan-Meier curves for cumulative probability of COVID-19 mortality during hospitalization in patients with different level of serum amylase. (**C**) Multivariable mixed-effect logistic regression model indicated that hyperamylasemia was independently associated with sepsis, DIC, Cardiac injury, ARDS and AKI.

Restricted cubic splines analysis was performed to flexibly model and visualize the association between serum amylase and all-cause mortality in COVID-19 patients. Regarding strong J-shaped association between serum amylase and all-cause mortality, the plot illustrated a substantial reduction of the risk within the lower range of serum amylase, which reached the lowest risk around 115 U/L and increased thereafter (*P* for non-linearity <0.001, Figure 2A). Above 115 U/L, the hazard ratio per standard deviation higher serum amylase calculated by mixed-effect Cox model was 2.85 (95% CI, 2.03 to 4.00).

In addition, mixed-effect Cox model was also constructed to assess the correlation between serum amylase and all-cause mortality. As a result, hyperamylasemia was highly associated to mortality in COVID-19 patients (Figure 2B, 1-3 times ULN: HR=2.29, 95% CI [1.49, 3.51], *P*<0.001; > 3ULN: HR=12.96, 95% CI [7.38, 22.76], *P*<0.001). After adjusting for age, gender, BMI, gastrointestinal symptoms, pre-existing comorbidities, and severity of COVID-19, hyperamylasemia remained an independent risk factor for mortality (1-3 times ULN: HR=1.63, 95% CI [1.04, 2.55], *P*=0.034; >3ULN: HR=8.90, 95% CI [4.96,15.97], *P*<0.001). In addition, based on multivariable mixed-effect logistic regression model, hyperamylasemia was identified to be independently associated with sepsis (Figure 2C, 1-3 times the ULN: OR=1.15, 95% CI [1.11,1.22], *P*<0.001; >3ULN: OR=1.87, 95% CI [1.64,2.15], *P*<0.001), DIC (1-3 times ULN: OR=1.13, 95% CI [1.10,1.17], *P*<0.001; > 3ULN: OR=1.65, 95% CI [1.50,1.81], *P*<0.001), cardiac injury(1-3 times ULN: OR=1.24, 95% CI [1.16,1.34], *P*<0.001; > 3ULN: OR=1.71, 95% CI [1.42,2.07], *P*<0.001), ARDS(1-3 times ULN: OR=1.21, 95% CI [1.14,1.28], *P*<0.001; > 3ULN: OR=1.62, 95% CI [1.38,1.92], *P*<0.001) and AKI(1-3 times ULN: OR=1.24, 95% CI [1.19,1.30], *P*<0.001; > 3ULN: OR=1.79, 95% CI [1.59,2.02], *P*<0.001).

### Associations between clinical characteristics and medications with serum amylase level

**Figure 3:**
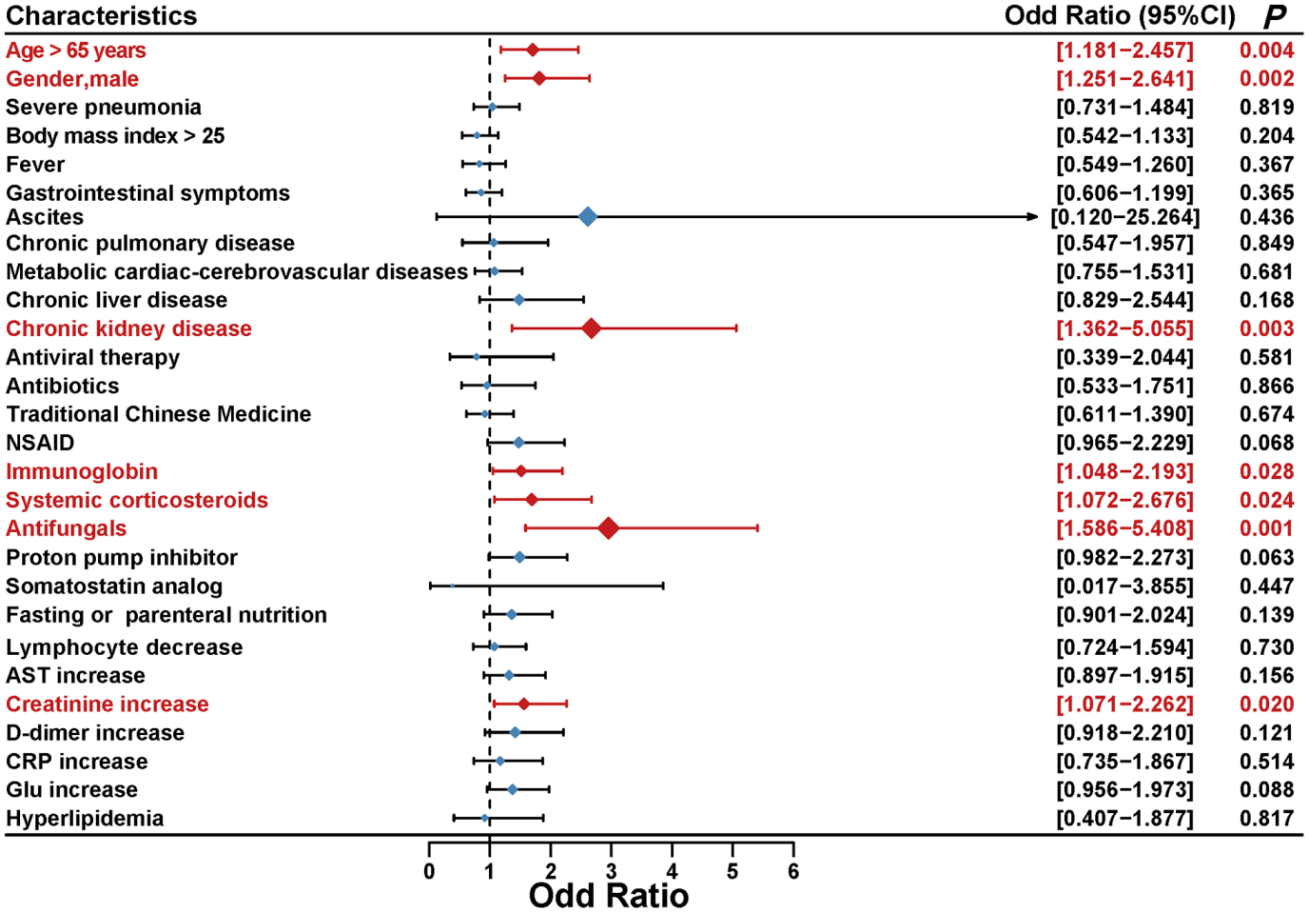
Multivariable ordered logistic regression analysis was performed to reveal the association between common clinical characteristics and peak amylase levels in COVID-19 patients.

With Cox regression model, ICR was calculate to assess the additive interaction between serum amylase and common clinical characteristics. The results indicated that there is no significance in additive interaction and no need to perform subgroup analysis to evaluate associations between clinical characteristics and serum amylase (Supplementary Figure 1). However, multivariable ordered logistic regression analysis revealed the effects of common clinical characteristics on peak amylase levels in COVID-19 patients from the longitudinal cohort, and the results indicated that older age, male, chronic kidney disease, several medications (immunoglobin, systemic corticosteroids, and antifungals) and increased creatinine might be independently associated with the elevated amylase levels during hospitalization in COVID-19 patients (Figure 3).

### Dynamic profile of serum amylase level in COVID-19 patients

**Figure 4:**
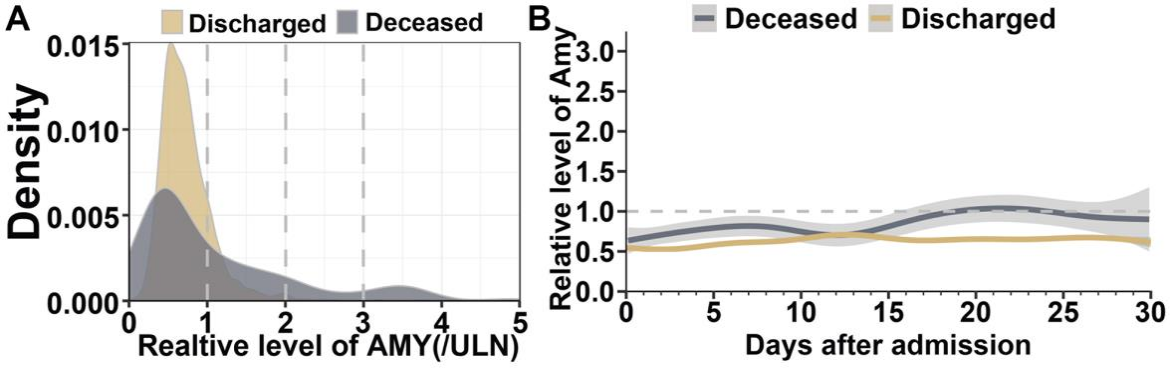
**Dynamic profile of serum amylase in patients with COVID-19 pneumonia.** (**A**) Kernel density estimates using Gaussian kernels to display serum amylase distributions between survived patients and those who died of COVID-19. (**B**) Smooth trajectories of mean amylase between survived and deceased patients with 95% confidence band based on GAM.

In order to ascertain distribution and trajectory of serum amylase in COVID-19 patients, multiple results from different days were recorded during hospitalization. By kernel density estimation, different serum amylase distributions were investigated between survivors and those who died of COVID-19. Amylase levels were lower and less disperse in survived cases, on contrast, the levels increased and grew more disperse in deceased patients (Figure 4A). GAM model was constructed to explicate dynamic trajectories of serum amylase in both survived and deceased cases during hospitalization (Figure 4B). The results indicated amylase level was higher in the deceased cases than in those who recover from COVID-19 infection. The fluctuation in survivors was mild and their amylase level predominantly maintained in the normal range. Nonetheless, the amylase level in deceased cases gradually increased, surpassed the ULN, and reached its peak within 18 to 22 days.

**Figure 5:**
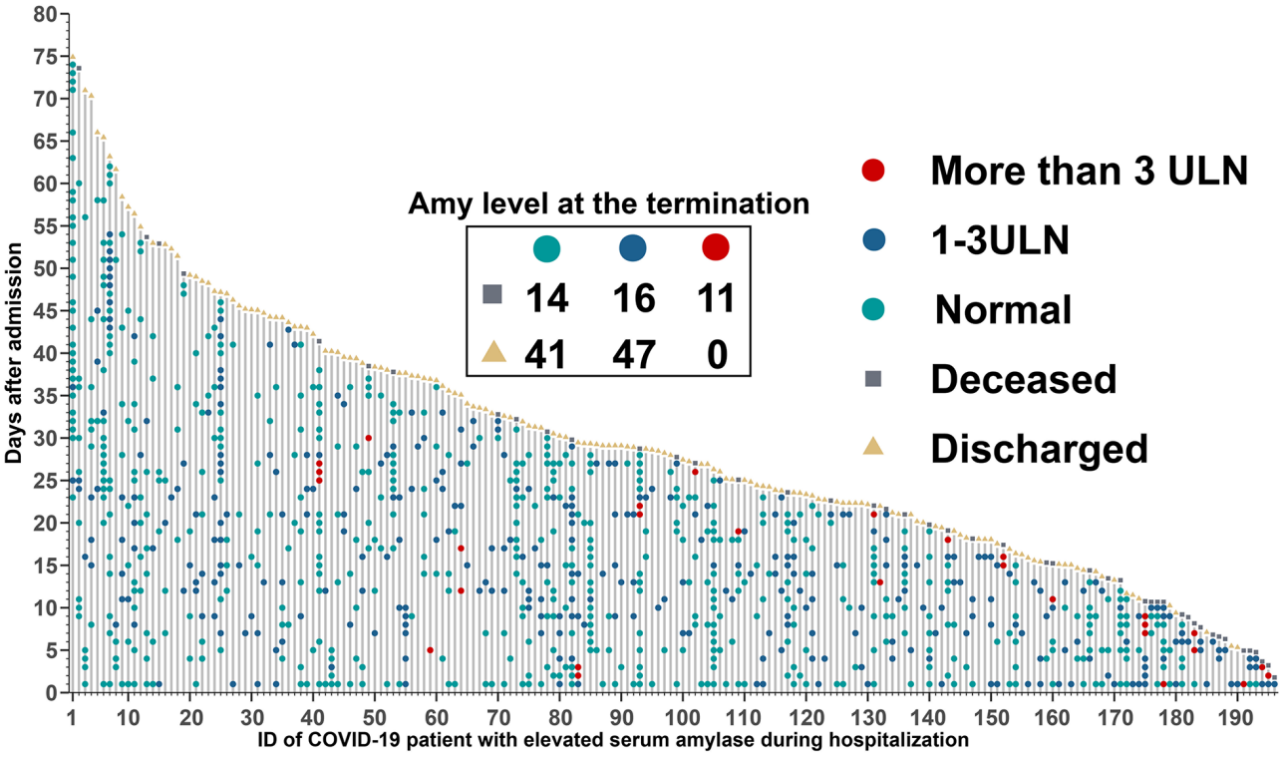
**Timeline of events for 196 COVID-19 patients with hyperamylasemia.** Amylase level at the termination time was extracted within 5 days before discharge or death, and 67 patients with no available data.

The timeline with death, discharge and different amylase levels marked (Figure 5), was performed to further assess the time-dependent development of hyperamylasemia events. Amylase level in the terminal time between survived and deceased patients were significantly different, which indicated that 11 (26.8%), 16 (39.0%), and 14 (34.1%) of 41 patients deceased with higher than 3 times ULN, 1-3 times ULN, and normal amylase level, separately, whereas, 0, 47 (53.4%), and 41 (46.6%) of 88 patients survived with higher than 3 times ULN, 1-3 times ULN, and normal level. This dynamic profile contributed to identify potential mechanisms of COVID-19-associated hyperamylasemia.

## DISCUSSION

Detrimental complication, along with multi-organ dysfunction, which evidently associated with in-hospital mortality, is not uncommon in COVID-19 patients [10]. Our study illustrated trajectories of serum amylase levels in hospitalized COVID-19 patients and portrayed their clinical significance based on comprehensive analysis in a large retrospective cohort of 1515 participants. The deceased cases manifested with greater fluctuation in serum amylase level, in contrast, the variation in survivors was mild and primarily maintained in the normal range. Our study first reported the association between serum amylase level elevation and in-hospital mortality in a large cohort with comprehensive analysis after adjusting for potential cofounders. Moreover, our study discriminated that older age, male gender, chronic kidney disease, several medications (NSAIDs, immunoglobin, systemic corticosteroids, and antifungals) and increased creatinine might be independently associated with the elevated amylase levels during hospitalization in COVID-19 patients.

A previous study reported 17% of COVID-19 patients presented with some form of pancreatic injury, based on any abnormality in serum amylase or lipase [11]. In the aforementioned study, of the all documented 52 COVID-19 patients, 13.5% (7/52) had elevated serum amylase, which share the similar incidence of serum hyperamylasemia with our data (12.9%). This retrospective study attributed hyperamylasemia to pancreatic injury. Nevertheless, it is cursory to concluded that SARS-COV-2 lead to mild or slight pancreatic injury, for higher serum levels of amylase can be caused by non-pancreatic etiologies, including intestinal disease [30], malignancy [30], acidosis [31], renal failure [32], and diabetes [32], other than pancreatic injury. On the other hand, a previous animal model indicated that epithelial cells lining salivary gland ducts were the early targets by the predecessor of SARS-CoV-2, SARS-CoV-1, which could contribute to serum amylase elevation other than pancreatic injury [33]. Besides, any physiological change, including elevated vascular permeability, disordered lymphatic drainage, and abnormal clearance would give rise to serum hyperamylasemia. Therefore, impaired renal excretion of amylase might be one of the major sources of hyperamylasemia in our cohort, which was similar to previous studies [34, 35].

The major COVID-19 treatment included antivirals, antibiotics, and systemic corticosteroids and antifungal was more likely to be administrated in weaker patients [36]. In our study, the results indicated that use of NSAIDs, immunoglobin, antifungal and systemic corticosteroids presented a positive associated with serum hyperamylasemia. Although these evidences could not directly infer the causal impact of drugs on hyperamylasemia, it is recommended that clinicians should pay more attention to the drug toxicity during the treatment of COVID-19 infection.

There is a discrepancy on the serum amylase testing between the health providers. Nevertheless, we discriminated no difference in the prevalence of gastrointestinal symptoms between patients with elevated serum and their counterparts of normal amylase level, which imply that they were not the primary factor that impelled clinicians to check the amylase in COVID-19 patients. Interestingly, our data seemed that gastrointestinal symptoms were not associated with serum amylase, but significantly more prevalent in individuals with amylase level more than 3 times ULN, the probability of hyperamylasemia resulted from colonic or enteric involvement of the virus as well as cannot be excluded. Further investigations including large number of patients with more than 3 times ULN amylase are needed to better understand the association between hyperamylasemia and gastrointestinal symptoms.

Although it was less specificity to diagnose pancreatic injury merely according to hyperamylasemia [16, 29], the probability of pancreatic damage cannot be eliminated. Due to high expression of angiotensin-converting enzyme-2 (*ACE2*) receptors and transmembrane serine protease 2 (*TMPRSS2*) in pancreatic tissue (Supplementary Figure 2), it is reasonable to suppose that the pancreas could be the attack target of SARS-CoV-2 [37, 38]. Moreover, this ratiocination was supported by a family cluster of acute pancreatitis associated with SARS-CoV-2 in Denmark [39], the detection of SARS-CoV2 RNA in a pancreatic pseudocyst fluid sample collected from a COVID-19 patient [40], autopsy specimen of COVID-19 patients who presented with slightly degeneration in pancreatic islet [41]. Hereby, whether the potential pancreatic injury caused by the direct attack by SARS-COV-2 or as part of the secondary hypoxemia, systemic inflammatory response and cytokine cascade leading to multi-organ damage. Nevertheless, clinical manifestations and limited radiological evidence in those who had a serum amylases above 3 ULN revealed that no diagnosis of acute pancreatitis could be established in our study. However, the probability of mild pancreatic injury that imaging examinations might not be discriminated could not be excluded. Consequently, serum amylase level elevation in COVID19 may be attributed to pancreatic injury, multiorgan-damaged involvement, as well as drug toxicity or multi-factor. Though, our results show that elevated amylase might be an independent risk factor of detrimental outcomes in COVID-19 infection.

Our study suffered several limitations. Firstly, due to its retrospective nature, the multiple tests for serum amylase were performed at different time intervals for each individual. Hence, bias might occur on the condition that when a patient’s condition deteriorates, more tests are done and usually get a worse result than in patients with a better course of illness. secondly, given the observational nature of our study, it could only demonstrate association, not causation. Whether hyperamylasemia is caused by SARS-CoV-2 needs to be further evaluated by direct clinical investigation. Last but not least, no measures were taken to distinguish analytically the distinct isoforms of serum amylase, so we could not reveal the real source of amylase (salivary or pancreatic amylase).

## CONCLUSIONS

The dynamic patterns of serum amylase and their potential risk factors might provide a crucial explanation for the COVID-19-related hyperamylasemia. Since hyperamylasemia is significantly associated with in-hospital mortality, it is crucial to monitor serum amylase vigilantly in COVID-19 patients.

## Supplementary Material

Supplementary Figures

Supplementary Table 1
