# Supplementary material for: Serum amylase elevation is associated with adverse clinical outcomes in patients with coronavirus disease 2019
Source: Aging (Albany NY). 2021 Oct 29;13(20):23442–58. doi: 10.18632/aging.203653 (PMC8580346; doi:10.18632/aging.203653)
Supplement: Supplementary Figures [file aging-13-203653-s001.pdf]

SUPPLEMENTARY FIGURES

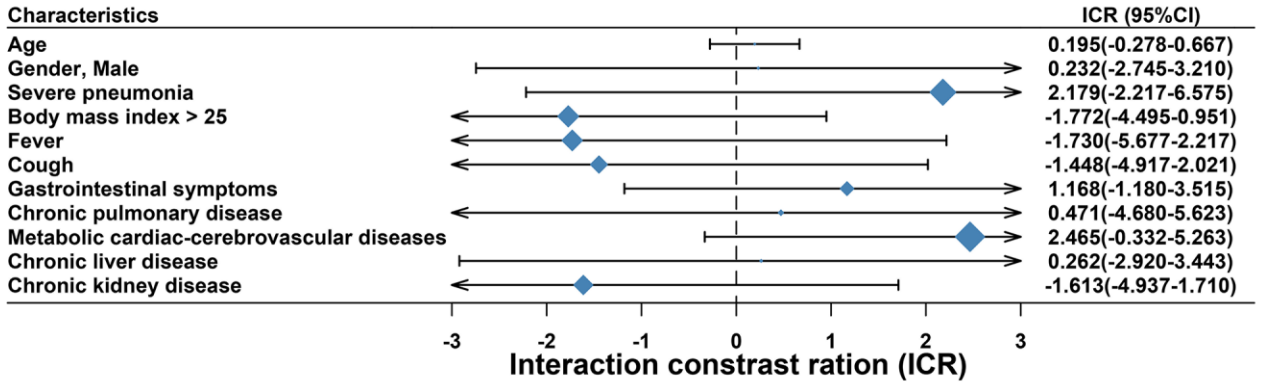

Supplementary Figure 1. Interaction contrast ratios (ICR) were calculated to assess the additive interaction between serum amylase and common clinical characteristics in the Cox regression.

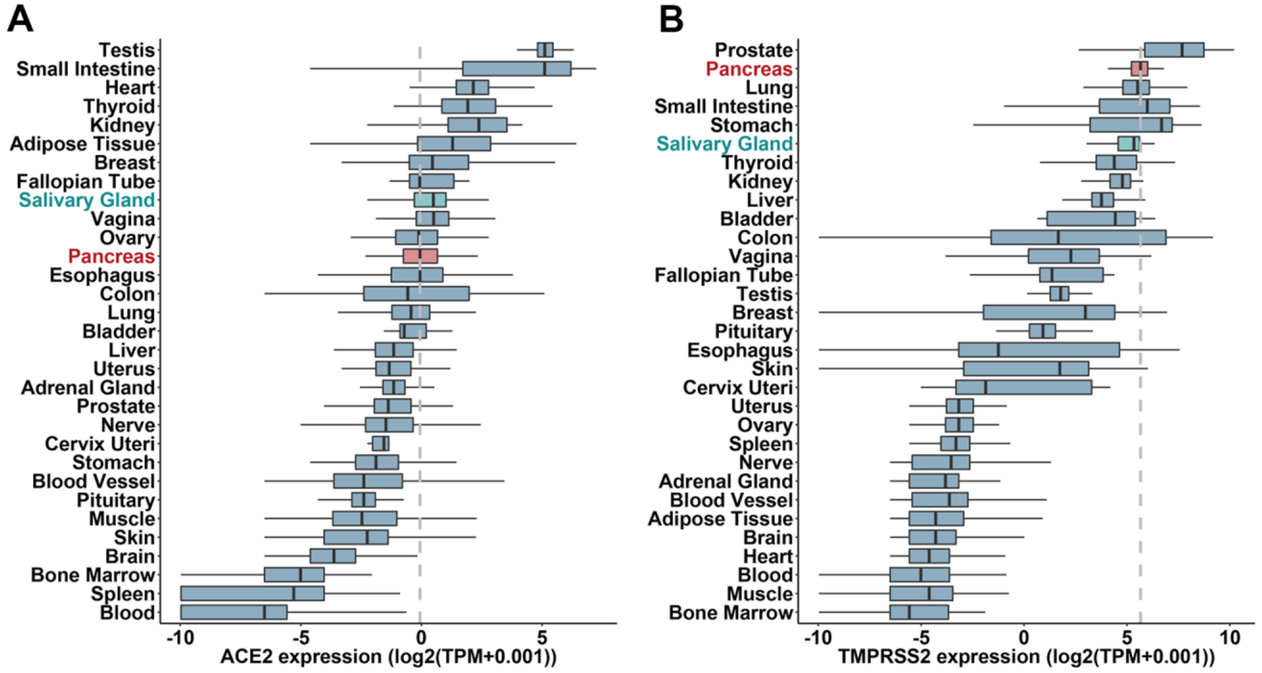

Supplementary Figure 2. The mRNA expression of *ACE2* (**A**) and *TMPRSS2* (**B**) in 31 normal human tissues from GTEx.
