# Supplementary material for: Serum amylase elevation is associated with adverse clinical outcomes in patients with coronavirus disease 2019
Source: Aging (Albany NY). 2021 Oct 29;13(20):23442–58. doi: 10.18632/aging.203653 (PMC8580346; doi:10.18632/aging.203653)
Supplement: Supplementary Table 1 [file aging-13-203653-s002.pdf]

SUPPLEMENTARY TABLE

Supplementary Table 1. The clinical characteristics of patients with 3 times ULN.

Characteristics	Above 3 folds of ULN (N=19)
Age- yr	69[64, 76]
Age≥ 65	14 (73.7)
Male	11 (57.9)
BMI	23.87 [23.47, 25.74]
Time from illness onset to hospital admission, days	10[7, 15]
Severe pneumonia*	14 (73.7)
Signs and symptoms	
Fever	13 (68.4)
Cough	14 (73.7)
Fatigue	4 (21.1)
Chest pain	1 (5.3)
Gastrointestinal symptoms**	10 (52.6)
Dyspnea	9 (47.4)
Myalgia	1 (5.3)
Administration of mechanical ventilation	15 (78.9)
Non-invasive	1 (5.3)
Invasive	14 (73.7)
Admission to intensive care unit	16 (84.2)
Renal replacement therapy	7 (36.8)
Extracorporeal membrane oxygenation	1 (5.3)
Clinical outcomes	
In-hospital death	15 (78.9)
Duration from illness onset to death, days	31[23, 36]
Hospital discharge	4 (21.1)
Duration from illness onset to discharge, days	41[37, 46]
Complications	
Acute kidney injury	13 (68.4)
Acute respiratory distress syndrome	15 (78.9)
Acute heart failure	16 (88.9)
Cardiac injury	16 (88.9)
Sepsis	15 (78.9)
Disseminated intravascular coagulation	11 (57.9)

1. Data were provided as number (percentage), median (interquartile range).
2. Serum Amylase Level, in a healthy individual, a normal blood amylase level ranges from 0-115 units per liter (U/L) in our hospital. Patients with serum amylase >345 U/L were classified into group of above 3 Folds of ULN (N=19).
3. Severe pneumonia*, the illness severity was classified according to Guidance for Corona Virus Disease 2019 (6/7th edition) released by the National Health Commission of China; Gastrointestinal symptoms**,including anorexia, nausea or vomiting, diarrhea, abdominal pain.
